# Sirtuins: Potential Therapeutic Targets for Defense against Oxidative Stress in Spinal Cord Injury

**DOI:** 10.1155/2021/7207692

**Published:** 2021-06-24

**Authors:** Jialiang Lin, Zhencheng Xiong, Jionghui Gu, Zhuoran Sun, Shuai Jiang, Dongwei Fan, Weishi Li

**Affiliations:** ^1^Department of Orthopaedics, Peking University Third Hospital, Beijing, China; ^2^Beijing Key Laboratory of Spinal Disease Research, Beijing, China; ^3^Engineering Research Center of Bone and Joint Precision Medicine, Ministry of Education, Beijing, China; ^4^Institute of Medical Technology, Peking University Health Science Center, Beijing, China; ^5^Peking University Third Hospital, Beijing, China; ^6^Department of Ultrasound, The First Affiliated Hospital, College of Medicine, Zhejiang University, Hangzhou, China

## Abstract

Spinal cord injury (SCI) is one of the most incapacitating neurological disorders. It involves complex pathological processes that include a primary injury and a secondary injury phase, or a delayed stage, which follows the primary injury and contributes to the aggravation of the SCI pathology. Oxidative stress, a key pathophysiological event after SCI, contributes to a cascade of inflammation, excitotoxicity, neuronal and glial apoptosis, and other processes during the secondary injury phase. In recent years, increasing evidence has demonstrated that sirtuins are protective toward the pathological process of SCI through a variety of antioxidant mechanisms. Notably, strategies that modulate the expression of sirtuins exert beneficial effects in cellular and animal models of SCI. Given the significance and novelty of sirtuins, we summarize the oxidative stress processes that occur in SCI and discuss the antioxidant effects of sirtuins in SCI. We also highlight the potential of targeting sirtuins for the treatment of SCI.

## 1. Introduction

Spinal cord injury (SCI) is a common central nervous system injury characterized by varying degrees of sensorimotor dysfunction, which can often lead to paraplegia, quadriplegia, and other pathologies that significantly affect the quality of life of a patient. The total global incidence of SCI has been estimated to be 3.6–195.4 cases per million people [1]. In the United States alone, the annual incidence of SCI is approximately 54 cases per million, with approximately 17,730 new cases of SCI occurring each year [2]. Due to its prevalence in young and middle-aged adults, predominantly in the age group of 35–64 years [3], SCI imposes a great economic and medical burden on society. Statistically, the lifetime cost of medical care and other injury-related expenses for a SCI patient is estimated to be 1.47–3.03 million dollars [4]. In addition to its impact on society, SCI also places a tremendous physical and psychological burden on the patients themselves, especially with the improvement of the survival rate of SCI patients in recent years. Methylprednisolone (MP) is currently the only FDA-approved drug recognized for the treatment of SCI. However, owing to the narrow window of administration time and numerous side effects, only a small proportion of SCI patients benefit from MP administration [5–7]. Therefore, there is an urgent need to identify new molecular target candidates and elucidate their cellular mechanism of action to develop new therapeutics for SCI.

Oxidative stress refers to an imbalance between oxidative and antioxidant cellular pathways in an organism, leading to the accumulation of excessive free radicals, including reactive oxygen species (ROS) and reactive nitrogen species, which in turn cause a series of cytotoxic effects. To date, oxidative stress has been demonstrated to play a central role in the pathogenesis of SCI [8–10]. Previous studies have shown that large amounts of ROS are generated immediately following SCI, which can induce oxidative stress if not neutralized promptly [11, 12]. More importantly, oxidative stress is associated with secondary events [13–16], such as inflammatory response, excitotoxicity, and neuronal and glial cell apoptosis after primary injury. Notably, the spinal cord is particularly vulnerable to peroxidation by ROS because of its high polyunsaturated fatty acid content [9]. Therefore, the spinal cord is highly susceptible to oxidative damage. The significance of ROS and lipid peroxidation during SCI has been validated by numerous experimental and clinical studies [17–22], and therapeutic strategies targeting oxidative stress pathways are increasingly showing promising applications.

Sirtuins, a conserved class of nicotinamide adenine dinucleotide (NAD)^+^-dependent protein deacetylases, are represented in mammals by seven member enzymes (SIRT1-7) [23, 24]. As the understanding of the function of the sirtuin family has improved, researchers have begun to focus on their antioxidant effects. Many studies have shown that sirtuins, particularly SIRT1 and SIRT3, are involved in cellular antioxidant defense mechanisms [25–27]. The redox signaling pathways regulated by sirtuins often are the ones that, when altered, play important roles in the occurrence and development of various pathologies [28–30], including SCI. This suggests that sirtuins are promising antioxidant enzymes that could be molecular targets for the treatment of SCI. In this review, we provide a synopsis of the involvement of oxidative stress in SCI and summarize the results from available literature, discussing the mechanisms and therapeutic approaches that target sirtuins to protect the spinal cord from oxidative stress-induced injury after SCI.

## 2. Pathophysiology of SCI

The pathophysiology of SCI involves two consecutive stages: a primary injury and a secondary injury [31, 32]. The primary injury refers to the stage of spinal cord damage that occurs immediately after the direct injury to the spinal cord, and it is usually the decisive element for the severity of SCI. Several common mechanisms can cause primary injury, including compression, contusion, shear, laceration, and acute stretching [33]. Generally, the spinal cord damage that constitutes primary injury is characterized by the disruption of neural parenchyma, shearing of the axonal network, destruction of the glial membrane, and vascular disruption [34]. The secondary injury is a delayed and prolonged pathological stage triggered by the primary injury, which aggravates the spinal cord tissue damage through a cascade of biological events [14, 15, 32, 35–41], including ischemia, vascular dysfunction, edema, excitotoxicity, formation of free radicals, glial and neuronal apoptosis, and inflammatory response. Currently, it has become evident that most of the posttraumatic degeneration of the spinal cord is caused by the secondary injury, which can occur during a period ranging from minutes to years after the primary injury, resulting in further damage to the surrounding tissues [42–44]. Therefore, neuroprotective interventions at the stage of the secondary injury, within an advisable “time window”, are essential to reduce cord damage and preserve neurological function.

The secondary injury involves pathophysiological processes that can be categorized into three contiguous phases that develop over time: the acute, subacute, and chronic phases [34, 42]. The acute phase, the dominant period in the secondary injury process, can be characterized by the pathophysiological processes of vascular disruption, continuous hemorrhage, and the resulting progressive ischemia and edema [32, 45, 46]. These pathophysiological events contribute to additional elements of the secondary injury cascade, including generation of free radicals, lipid peroxidation, inflammation, ionic dysregulation, excitotoxicity, and apoptosis and necrosis of neurons [14, 16, 47–50]. After the primary injury, the damage to the microcirculation leaves the adjacent tissues in a state of hypoperfusion, and the resulting ischemia and hypoxia further lead to the swelling of neurons and glial cells, blocking the conduction of action potentials [51]. Excitotoxicity is mainly caused by excessive activation of glutamate receptors. Following injury, extracellular glutamate can reach excitotoxic levels within a few minutes, which contributes to the influx of Ca^++^ and Na^+^ [16, 52]. Subsequently, high levels of intracellular Ca^++^ trigger a series of destructive events, including free radical formation, which ultimately leads to neuronal cell death [48]. In addition, the activation of microglia begins almost immediately after injury, along with an increase in proinflammatory cytokines such as TNF-*α* and IL-*β*, which can be detected a few minutes after the injury [53, 54]. In the subacute phase, apoptosis of oligodendrocytes is a significant pathological feature [55], with apoptotic oligodendrocytes fragmenting into apoptotic bodies, which are subsequently cleared by phagocytosis. The phagocytic response is most evident during the subacute phase. It may contribute to the removal of apoptotic fragments from the lesion and can somewhat promote the growth of axons by removing the myelin debris [53]. Over time, various proinflammatory and anti-inflammatory mediators peak one after another during this period [56], which constitutes another important hallmark of this phase. On the one hand, inflammation can remove cellular debris and provide a favorable environment for tissue repair and regeneration; on the other hand, excessive activation of inflammatory cascades can exacerbate the damage [13]. Thus, inflammation should be regarded as a double-edged sword, which possesses both neuroprotective and neurotoxic properties [46]. Furthermore, inflammation and oxidative stress are closely related and interacting with pathological processes during SCI. It is critical to gain insight into the characteristics of the inflammatory response and to delineate beneficial and deleterious aspects of it to target them therapeutically. In addition, astrocytes around the lesion begin to proliferate and become hypertrophic during the subacute phase, forming a gliotic scar. Apart from scar formation, astrocytes also play a critical role in maintaining ionic homeostasis and restoring the integrity of the blood-brain barrier after SCI [57]. The chronic phase of SCI is characterized by the maturation of the lesion, which eventually forms a cavity surrounded by glial and fibrotic scars. However, up to 30% of SCI patients have spinal cord cavities that remain progressive during the chronic phase, leading to delayed neurological damage and neuropathic pain [36, 58].

ROS production and apoptosis are crucial processes in the pathophysiology of SCI, as described in the following section.

## 3. Oxidative Stress in SCI

### 3.1. Production and Elimination of Reactive Oxygen Species

Previous studies have shown that a large number of free radicals are generated after SCI, represented by ROS, which are important contributors to secondary damage [11, 12]. It is well known that ROS are the normal by-products of oxygen metabolism, and include superoxide, hydroxyl radicals, singlet oxygen, and hydrogen peroxide [59]. Under physiological conditions, low intracellular concentrations of ROS facilitate the maintenance of cellular homeostasis by stimulating endogenous antioxidant defense mechanisms and enhancing cellular repair processes [60]. However, excessive production of ROS can overwhelm the antioxidant defenses and can have lethal effects on cells by damaging vital cellular components such as lipids, proteins, and nucleic acids. For example, ROS can induce lipid peroxidation, which tends to attack and degrade polyunsaturated lipids [61]. These lipids are essential components of biological membranes, and their disruption results in cellular dysfunction, ultimately leading to cell death. Meanwhile, ROS are known to react with the components of proteins, including cleavage of the polypeptide chain, directed protein degradation, and amino acid side chain modifications [62]. Additionally, ROS-induced oxidation of DNA can cause a range of reactions, such as disruption of the purine and pyrimidine bases [63].

Mitochondria are the “powerhouse” of cells, utilizing approximately 90% of intracellular oxygen by oxidative phosphorylation; meanwhile, mitochondria are also the major source of intracellular ROS [64]. In the pathogenesis of SCI, ROS generation is closely associated with postinjury ischemia and secondary reperfusion injury [49]. More importantly, Wingrave et al. [65] found that impairment of mitochondrial structure and function occurred in a rat model of SCI, which further led to substantial ROS formation. Elevated ROS can cause cell membrane damage and organelle dysfunction via lipid peroxidation, which then leads to a cascade of secondary injury events, including disruption of calcium homeostasis and release of excitatory amino acids. All these events, in turn, may further lead to mitochondrial dysfunction and increased ROS production, resulting in a vicious cycle that ultimately leads to neural cell death [8, 66]. In addition to mitochondria, ROS may also originate from other organelles and cellular compartments [67, 68] such as peroxisomes, lysosomes, and the endoplasmic reticulum, or from the action of cytosolic oxidases.

There are several endogenous antioxidant defense mechanisms, including enzymatic and nonenzymatic antioxidants, that maintain cellular homeostasis. Specific enzymatic antioxidants primarily include superoxide dismutase (SOD), catalase (CAT), and glutathione peroxidase. These enzymes play a crucial role in cellular antioxidant defense. For instance, SOD exerts its antioxidant effects by converting superoxide to hydrogen peroxide; the decomposition of hydrogen peroxide can then be accomplished by catalase or glutathione peroxidase [59, 69]. Nonenzymatic antioxidants mainly include vitamin C and E, glutathione, and flavonoids [70]. All these antioxidant defense mechanisms ensure that cells scavenge the right amount of ROS to maintain cellular homeostasis under physiological conditions. However, when excess ROS exceed their own scavenging capacity, the cells will suffer from oxidative stress damage.

### 3.2. Oxidative Stress and Apoptosis

Apoptosis, or programmed cell death, is a controlled and energy-consuming process that occurs in multicellular organisms [47]. Numerous studies have shown that apoptosis is one of the major pathological manifestations of secondary injury [55, 71–73], and its severity directly affects the recovery of motor function in SCI patients to a large extent. Therefore, the inhibition of neuronal and glial apoptosis during secondary injury is a priority in the treatment of SCI. Liu et al. found that apoptosis of neurons occurs predominantly in the early stages of SCI and gradually decreases thereafter [14]; oligodendrocytes are the main cell population that undergoes apoptosis between 24 h and 3 weeks after SCI [73, 74]. A growing amount of evidence suggests that oxidative stress is closely associated with neuronal and glial apoptosis [15]. During the secondary damage phase of SCI, high levels of ROS induce lipid peroxidation of biological membranes. 4-hydroxynonenal, a lipid peroxidation product, has been found to accumulate after experimental SCI and to induce apoptosis when added to cultures of PC-12 cells or hippocampal neurons *in vitro* [72, 75]. Therefore, attenuating apoptosis during the period of secondary injury by suppressing excessive oxidative stress deserves further investigation.

## 4. Role of Sirtuins in Oxidative Stress

### 4.1. Distribution and Function of Sirtuins

The protein encoded by the silent information regulator 2 (Sir2) gene was first discovered in yeast and is believed to have the function of activating telomerase and ribosomal DNA, prolonging lifespan [26]. Sir2 homologs found in humans and mammals are named sirtuins, which are histone deacetylases with NAD^+^-dependent properties [76]. Sirtuins, although homologous to Sir2, differ in their N- and C-terminal structural domains, which usually consist of a conserved catalytic domain and variable N- and C-terminal structural domains [76]. The sirtuin family proteins (SIRT1-7) are localized in the nucleus, cytoplasm, and mitochondria, and they are expressed in multiple organs and tissues in humans and mammals [77]. The diversity of subcellular localization affects the functions of the sirtuin family proteins in cells. According to the molecular analysis of the conserved core domain sequence of sirtuins from various organs and tissues, the seven members of the sirtuin family are divided into four groups: SIRT1, SIRT2, and SIRT3 as class I; SIRT4 as class II; SIRT5 as class III; and SIRT6 and SIRT7 as class IV [26]. Among them, SIRT1 is the most widely studied member of the sirtuin family, owing to its crucial role in many biological processes including oxidative stress response, cellular metabolism, glucose homeostasis, and insulin secretion [26, 78, 79]. The human SIRT1 gene is on chromosome 10 and encodes a protein consisting of 746 amino acids that contains the NAD-binding catalytic core domain [76]. SIRT1 is expressed in a variety of tissues and cells *in vivo*, including the central nervous system, cardiomyocytes, hepatocytes, glomerular cells, and skeletal muscles [26]. Under physiological conditions, SIRT1 is present in the nucleus and cytoplasm and acts mainly in the nucleus to deacetylate transcription factors such as peroxisome proliferator-activated receptor-*γ* coactivator-1*α* (PGC-1*α*), p53, forkhead box O (FOXO) s, and nuclear factor-kappa B (NF-*κ*B) [76]. SIRT2 is an NAD^+^-dependent deacetylase, and the human SIRT2 gene, composed of 18 exons, is on chromosome 19 at q13 [77]. The SIRT2 protein is found in the cytoplasm and enters the nucleus during the G2/M transition, affecting the cell cycle [80]. SIRT2 is widely expressed in different organs and tissues, and actively participates in antioxidant- and redox-mediated cellular homeostasis [77]. SIRT2 possibly plays a role in cancer; however, it is unclear whether it acts as a tumor suppressor or as an oncogene. SIRT3, SIRT4, and SIRT5 enzymes are mainly located in the mitochondria where they regulate mitochondrial metabolism, energy production, and the formation of ROS to help maintain metabolic homeostasis [76]. SIRT3 possesses NAD^+^-dependent deacetylase activity, and the human SIRT3 gene is located on chromosome 11 at p15.5 [81]. SIRT3 is mainly distributed in mitochondria-rich tissues and organs, such as kidneys, brain, heart, and liver, and it is relatively less abundant in the testis, lung, ovary, and thymus [82]. The deacetylase activity of SIRT3 is expressed upon cleavage by mitochondrial processing peptidase [83]. In recent years, as the number of SIRT3-related studies has increased, the role of SIRT3 in the regulation of mitochondrial respiratory function, redox homeostasis, insulin response, metabolic adaptation, and stem cell differentiation has been demonstrated [84]. In addition to its deacetylase activity, SIRT4 mainly acts as an ADP-ribosyl transferase, primarily in the kidneys, liver, heart, testis, and skeletal muscle, where SIRT4 plays a regulatory role in mitochondrial function, antioxidant defense, lipid metabolism, and insulin secretion [85]. SIRT5 is a mitochondrial sirtuin that regulates mitochondrial respiration and redox homeostasis and has been found to have enzymatic activities such as deacetylase, desuccinylase, and demalonylase. SIRT5 is capable of removing acetyl, succinyl, and malonyl groups from the lysine residues of proteins [86]. The human SIRT6 gene is located on chromosome 19 and encodes a protein, known to be a nuclear protein, that deacetylates histone H3 lysine 9 and lysine 56 [87]. Studies have shown that SIRT6 plays a key role in human telomere and genome stability, oxidative stress, inflammation, and metabolism of glucose and lipids [25, 88]. Knockout of the SIRT7 gene in mice induces multisystemic mitochondrial dysfunction, as well as reduced lifespan [89]. SIRT7 is a nuclear protein that regulates RNA polymerase 1-mediated transcription with highly selective histone deacetylase activity, which plays a key role in chromatin regulation, tumor formation, and cellular transformation programs [90].  Table 1 shows the enzymatic activity, intracellular distribution, and potential mechanisms of action of the sirtuin family proteins relevant for SCI.

### 4.2. Sirtuins-Mediated Antioxidant Defense

#### 4.2.1. SIRT1 and SIRT2

**Figure 1:**
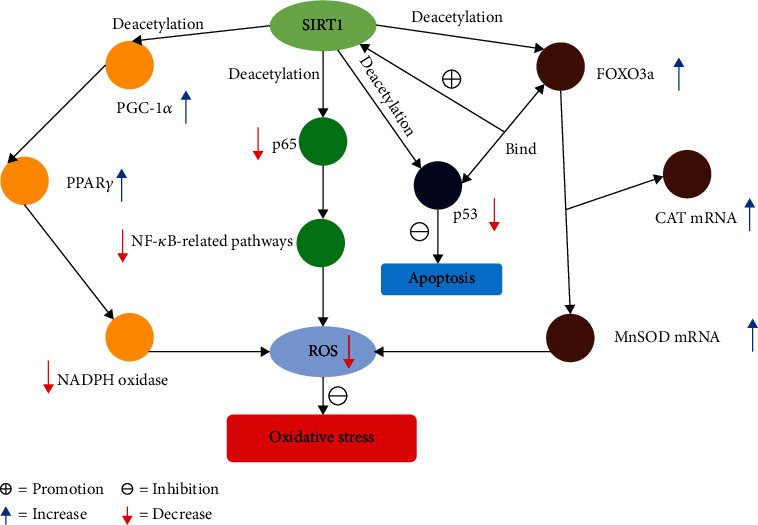
SIRT1 regulates different target genes against oxidative stress. The oxidative stress resistance of SIRT1 is partly attributable to its deacetylation of multiple targets, including PGC-1*α*, p53, FOXO3a, and NF-*κ*B. Among them, FOXO3a interacts with p53, forming a complex that promotes the binding of p53 and SIRT1, stimulates SIRT1 expression, and inhibits apoptosis and oxidative stress. (ROS: reactive oxygen species; CAT: catalase; MnSOD: manganese superoxide dismutase).

The antioxidant defense activity of SIRT1 is mostly due to its deacetylation of multiple targets, including PGC-1*α*, p53, FOXOs, and NF-*κ*B (Figure 1). Qin et al. [91] found that resveratrol could inhibit oxidative stress and apoptosis through the SIRT1/FOXO3a and PI3K/AKT pathways, thereby reducing radiation-induced intestinal injury. Mammalian FOXOs belong to the O class of the FOX transcription factor superfamily, which includes four members: FOXO1, FOXO3, FOXO4, and FOXO6 [92]. FOXOs play an important role in a variety of physiological processes, including cell cycle, apoptosis, oxidative stress protection, and homeostasis maintenance [93]. SIRT1 activates the transcriptional activity of some members of the FOXO family of proteins to exert protective effects against oxidative stress, maintain blood glucose homeostasis, and suppress inflammation. SIRT1 also inhibits the transcriptional activity of those FOXO genes involved in apoptosis, thus protecting the cells from apoptosis [94]. Brunet et al. [95] found that oxidative stress induced the translocation of FOXO3 from the cytoplasm to the nucleus, and in turn, SIRT1 deacetylated FOXO3, ultimately promoting cell survival. FOXOs can also regulate SIRT1 transcription by binding to SIRT1 promoter elements, upregulating SIRT1 mRNA expression and protein levels, and protecting against oxidative stress and aging-related diseases [96]. It was also shown that the SIRT1 activator resveratrol prevents cell death by reducing oxidative stress and protecting mitochondrial function in neuronal cell lines [97]. PGC-1*α* is a transcriptional coactivator that interacts with the nuclear receptor PPAR-*γ* to regulate genes involved in energy metabolism and is a major regulator of mitochondrial biogenesis [98]. Chen et al. [99] found that SIRT1 plays an important role in the maintenance of cellular redox homeostasis by deacetylating PGC-1*α*, activating and inducing its expression, promoting mitochondrial biosynthesis, increasing the level of antioxidant enzymes, and inhibiting the NADPH oxidase *in vivo*. The tumor suppressor p53 requires posttranscriptional modifications, including phosphorylation and acetylation, for its activation, and these modifications occur in a variety of stress situations [100]. Oxidative stress increases p53 nuclear translocation and enhances DNA binding capacity and transcriptional activity, leading to cell cycle arrest or apoptosis [91]. Kume et al. [100] demonstrated that SIRT1 inhibits the activity of p53 by deacetylating p53, thereby preventing apoptosis induced by oxidative stress. NF-*κ*B is a key protein that regulates inflammatory responses [101]. In the acute phase of inflammation, mitochondria produce excess ROS that further activate NF-*κ*B to induce the expression of proinflammatory mediators, exacerbating the inflammatory response and resulting in damage to the organism [102]. SIRT1 inhibits the NF-*κ*B signaling pathway by deacetylating p65, a subunit of NF-*κ*B, to alleviate inflammatory responses and oxidative stress [103]. In SIRT1 knockout embryonic stem cells, H_2_O_2_-induced oxidative stress leads to elevated expression of the apoptotic genes BAX and PUMA. SIRT1 reduces mitochondrial damage and protects cells from apoptosis by positively regulating autophagy [104]. The expression of SIRT1 is downregulated by *miR-182-5p*, which was identified as a direct target of *circ*ERCC2. Xie et al. [105] found that *circ*ERCC2 responds to oxidative stress by targeting the *miR-182-5p*/SIRT1 axis, significantly activating mitophagy, reducing degradation of the extracellular matrix, and inhibiting apoptosis. This result shows that SIRT1 expression plays a role in oxidative stress by regulating apoptosis and mitophagy. The findings above also further validate that the mechanism of SIRT1 as an antioxidant may be attributed to its deacetylation of multiple targets (including PGC-1*α*, p53, FOXOs, and NF-*κ*B), although the process is also regulated by miRNAs and other noncoding RNAs.

It has been reported that ROS production is a significant trigger for SIRT2 upregulation [77] and that SIRT2 activity is involved in cellular responses to oxidative stress [106]. Qu et al. [107] demonstrated that the inhibition of SIRT2 accelerates the development of diabetic osteoarthritis (OA), and the upregulation of SIRT2 alleviates the development of diabetic OA by inhibiting oxidative stress and inflammation, which may be related to histone H3 deacetylation. However, Kaitsuka et al. [108] found that SIRT2 inhibition induces VEGF, hypoxia-inducible factor-1*α* (HIF-1*α*), and heme oxygenase-1 gene expression and protects neuronal viability from oxidative stress. The contrasting results suggest that additional studies are needed to investigate whether it is the inhibition or the upregulation of SIRT2 that confers a protective effect on oxidative stress, or whether there are tissue-specific differences in SIRT2 function.

#### 4.2.2. SIRT3, SIRT4, and SIRT5

The mitochondrial sirtuins SIRT3, SIRT4, and SIRT5 act as key regulators of mitochondrial metabolism, oxidative stress, and cell survival. Several studies have demonstrated that SIRT3, through its deacetylation activity, can activate glutamate dehydrogenase in amino acid metabolism [109]; long-chain acyl-coenzyme A dehydrogenase in fatty-acid oxidation [110]; succinate dehydrogenase and isocitrate dehydrogenase 2 (IDH2) in the tricarboxylic acid cycle [109, 111]; and NADH dehydrogenase, ATP synthase, and acetyl-CoA synthetase 2 in the electron transport chain of oxidative respiration [112–114]. These enzymatic activities enhance mitochondrial oxidative respiration, ensuring the stability of mitochondrial energy metabolism and reducing ROS production. SIRT3 promotes ROS scavenging in mitochondria by enhancing the activities of various enzymes in the antioxidant system. Studies have shown that SIRT3 deacetylates and activates manganese SOD (MnSOD) in mitochondria and reduces ROS levels in response to oxidative stress [115]. In addition, SIRT3 deacetylates and activates mitochondrial IDH2, leading to elevated NADPH levels and an increased ratio of reduced glutathione to oxidized glutathione in mitochondria, thereby enhancing the mitochondrial glutathione antioxidant defense system [116]. It has also been shown that SIRT3 can deacetylate acetylated FOXO3a and increase the mRNA expression level of FOXO3a-dependent CAT, reducing the level of intracellular ROS to protect the heart [117]. Therefore, it can be suggested that SIRT3 can alleviate ROS-induced oxidative stress by directly or indirectly increasing the activities of ROS-scavenging enzymes such as MnSOD and CAT and by increasing the intracellular levels of reduced glutathione. All these results suggest that SIRT3, through its deacetylation activity, may regulate a variety of enzymes, transcription factors, and biological factors that play an important role in the regulation of oxidative stress processes.

A previous study suggested that overexpression of SIRT4 contributes to the inhibition of inflammatory responses and oxidative stress during OA and that SIRT4 could be a target for OA therapy [118]. However, Luo et al. [119] obtained contrasting results, as they found that SIRT4 promotes oxidative stress in angiotensin II-induced myocardial hypertrophy by increasing ROS levels and inhibiting SIRT3-mediated deacetylation of MnSOD, thereby promoting hypertrophic growth, fibrogenesis, and cardiac dysfunction. Therefore, it is still unclear whether SIRT4 activity exerts a beneficial role in decreasing oxidative stress levels, and further studies will be needed to explore SIRT4 function on redox homeostasis.

SIRT5 plays an important role in inhibiting peroxisome-induced oxidative stress, protecting the liver, and inhibiting the occurrence of hepatocellular carcinoma [120]. Ye et al. [121] found that SIRT5, because of its effect of stimulating cell proliferation and tumor growth in response to oxidative stress, could be a potential target for clinical cancer research. Zhou et al. [122] demonstrated that SIRT5 regulates cellular NADPH homeostasis and redox potential by promoting IDH2 desuccinylation and glucose-6-phosphate dehydrogenase deglutarylation. The current findings demonstrate that SIRT5 may be involved in oxidative homeostasis and tumor development by regulating oxidative stress processes.

#### 4.2.3. SIRT6 and SIRT7

Several studies have demonstrated the potential antioxidant activity of SIRT6 and SIRT7 toward different forms of oxidative stress [87, 123–128]. Huang et al. [87] found that the inhibition of SIRT6 increased the levels of inflammatory mediators and ROS, aggravating inflammation and oxidative stress, thus exacerbating diabetic cardiomyopathy. Collins et al. [123] demonstrated that by regulating specific members of the peroxiredoxin catalytic cycle, SIRT6 could maintain chondrocyte redox homeostasis. Zhou et al. [124] demonstrated that SIRT6 protects from acetaminophen-induced hepatotoxicity by reducing oxidative stress and promoting hepatocyte proliferation. Knockdown of FOXO6 enhances nuclear factor erythroid 2-related factor 2 (Nrf2) activation through upregulation of SIRT6, protecting cardiomyocytes from hypoxia-induced apoptosis and oxidative stress [125]. The results above suggest that SIRT6 may play a significant role in redox homeostasis by inhibiting oxidative stress and inflammation.

Vakhrusheva et al. [126] found that SIRT7-deficient primary cardiomyocytes showed a significant increase in basal apoptosis, suggesting a key role of SIRT7 in regulating oxidative stress and cell death in the heart. The mechanism for the cardioprotective effect of SIRT7 may be due to the fact that SIRT7 increases resistance to cytotoxicity and oxidative stress by deacetylating p53. Lewinska et al. [127] found that vascular smooth muscle cells exhibited SIRT7 downregulation and increased p53 stability in response to curcumin-induced oxidative damage. The mechanism for this effect may be that SIRT7 downregulation reduces RNA polymerase 1-mediated transcription and stabilizes p53 to activate the target protein p21, which ultimately leads to cell cycle arrest. HIF is an important transcription factor that mediates adaptation to hypoxia [128]. The mechanism by which SIRT7 regulates HIF activity differs from that of other sirtuins because SIRT7 downregulates HIF at the protein and transcriptional level in a way that is independent of deacetylase activity [128]. The above results indicate SIRT7 may play a regulatory role in oxidative stress by regulating P53 stability and HIF activity.

## 5. Targeting Sirtuins for Potential Therapeutic Applications in SCI

### 5.1. Therapeutic Potential of Targeting SIRT1 in SCI

SIRT1 is widely distributed throughout the brain and spinal cord in rodents and humans, with subcellular localization predominantly in the nucleus [129]. Numerous studies have shown that SIRT1 plays a key role in the central nervous system (CNS) by regulating various intracellular activities [30, 130–146]. Resveratrol, a classical activator of SIRT1, has been reported to inhibit apoptosis of VSC4.1 motor neurons by promoting SIRT1-mediated autophagy [130]. Zhao et al. [131] found that resveratrol could inhibit neuronal apoptosis in SCI rats, reduce tissue damage, and promote recovery of motor function by activating autophagy mediated by the SIRT1/AMPK signaling pathway. Moreover, many studies have shown that resveratrol is beneficial in various *in vitro* and *in vivo* models of neuronal death and degeneration in the CNS. However, Tang et al. suggested that the neuroprotective effects of resveratrol may not be directly mediated by SIRT1, but more likely by AMPK [132]. AMPK is an enzyme that plays a role in cellular energy homeostasis, largely activating the uptake and oxidation of glucose and fatty acids when cellular energy is scarce. AMPK also regulates the initiation of autophagy, exerting antioxidant effects. Studies have shown that AMPK is involved in the prevention of oxidative stress due to the activation of SIRT1 and FOXO1 [133]. Similar results demonstrating the protective effects of AMPK/SIRT1-mediated autophagy on spinal cord neurons were also reported by Yan et al. [134]. Resveratrol has also been shown to activate the SIRT1-mediated sonic hedgehog signaling to exert antioxidant and anti-inflammatory effects to inhibit fibrous scar formation after SCI [135]. Furthermore, resveratrol protects the lung from SCI-induced inflammatory damage by upregulating SIRT1 expression and inhibiting NF-*κ*B activity, making it a treatment option for lung disease occurring after SCI [136]. Additionally, melatonin also exerts neuroprotective effects on SCI by activating autophagy and inhibiting apoptosis via the SIRT1/AMPK signaling pathway [137]. Chen et al. found that SRT1720, a SIRT1 agonist, contributed to improved outcomes after SCI in wild-type mice by inhibition of inflammation; however, SIRT1-knockout mice exhibited worse locomotor recovery [138]. Notably, MLN4924, a potent inhibitor of the NEDD8-activating enzyme, significantly attenuates oxidative stress and neuronal cell death by regulating SIRT1 expression during spinal cord ischemia-reperfusion injury [30]. Further experiments are needed to verify whether resveratrol directly activates SIRT1 to exert neuroprotective effects; however, it is clear that SIRT1 plays an important role in neuroprotection after SCI.

Several miRNAs have been reported to be potential therapeutic targets for SCI by regulating SIRT1. Yu et al. [139] found that SIRT1 contributes to the inhibition of apoptosis via the p53 signaling pathway in SCI, both *in vivo* and *in vitro*, whereas *miR-494* inhibits this process and induces apoptosis by targeting SIRT1. In addition, *miR-138-5p* has been reported to play important roles in the development of SCI by regulating the PTEN/AKT signaling pathway via SIRT1 [140]. Wang et al. [141] demonstrated that the depletion of *miR-30c* protects PC-12 cells from apoptosis and inflammation caused by oxygen-glucose deprivation through targeting SIRT1, thereby mitigating spinal cord ischemia-reperfusion injury. Moreover, downregulation of *miR-448* inhibits neuronal apoptosis and improves neurological function by upregulating SIRT1 to reduce spinal cord ischemia-reperfusion injury [142]. Consequently, more miRNAs targeting SIRT1 should be investigated and developed as potential strategies to treat SCI.

Microglia, with their polysynaptic and plastic characteristics, are intrinsic immune effector cells in the CNS and release a variety of cytotoxic substances in acute neurodegenerative diseases that directly damage neurons and lead to neuronal death in the CNS [143]. Thus, microglia activation is a key factor in posttraumatic inflammation and oxidative stress. Consequently, regulation of microglia activation is crucial for the recovery of neuronal function. Lu et al. [144] demonstrated that SIRT1 exerts neuroprotective effects by downregulating Wnt/*β*-catenin signaling to inhibit microglia activation, thereby reducing local inflammation and cellular stress in the early stages of SCI. Astrocytes also play important roles in the repair and reconstruction of SCI. The SIRT1/Nrf2 pathway in astrocytes can be activated by NFAT5, which exerts antioxidative stress effects against oxygen-glucose-serum deprivation/restoration damage [145]. Notably, reduced SIRT1 is also one of the injury mechanisms causing impairment in CNS energy homeostasis after SCI in a Western diet, which is linked to astrocyte metabolism [146]. These results confirm the crucial roles of SIRT1 during the pathological process of SCI and highlight SIRT1 as an effective target for the treatment of SCI.

### 5.2. Therapeutic Effects of Targeting Other Sirtuins for SCI

Many studies have explored the roles of six sirtuins other than SIRT1 for the treatment and functional recovery of SCI [108, 147–155]. SIRT2 promotes the differentiation of ependymal stem cells into neurons after SCI by increasing the deacetylation of stable Ac-*α*-tubulin in microtubules to improve neural recovery [147]. However, Kaitsuka et al. [108] demonstrated that SIRT2 inhibition activates HIF-1*α* signaling and protects neuronal viability from oxidative stress. This opposite conclusion regarding SIRT2 may be related to the different time points of its role in SCI pathophysiology. Song et al. [148] found that SIRT3 and PGC-1*α* protect rat spinal cord motor neurons from mutant SOD1(G93A)-induced mitochondrial fragmentation and neuronal cell death by maintaining mitochondrial dynamics. Recently, SIRT4 was shown to inhibit the antineuroinflammatory activity of regulatory T cells infiltrating the traumatically injured spinal cord by suppressing the AMPK signaling pathway [149]. SIRT5 plays a major role in PKC*ε*-mediated neuroprotection against cortical degeneration and neural cell death following cerebral ischemia [150]. However, SIRT5 has both antiapoptotic and proapoptotic effects. Subcellular localization may be a significant determinant of the effect of SIRT5 on neuron viability [151]. Furthermore, SIRT6 also acts as a protective factor against SCI by inhibiting inflammation, oxidative stress, and cell apoptosis to attenuate damage [152]. However, Shao et al. [153] showed that SIRT6 enhanced the damage induced by oxidative stress in neuronal cells, which was related to necrotic cell death and increased ROS production. In addition, SIRT7 may protect neurons from oxygen-glucose deprivation and reoxygenation-induced damage by regulating the p53-mediated proapoptotic signaling pathway [154]. The protective effects of low concentrations of ferulic acid on PC12 cells against H_2_O_2_-induced apoptosis were partially mediated by SIRT7 [155]. The above research results indicate that sirtuins are promising potential targets for the prognosis and treatment of SCI. However, in order to better apply therapeutic approaches targeting sirtuins in SCI, it is necessary to elucidate the distinct roles of sirtuins in different pathophysiological phages and the appropriate time window for therapeutic intervention.

## 6. Concluding Remarks

The research discussed herein supports that oxidative stress and its products can damage important macromolecules, such as lipids, proteins, and DNA, disrupting intracellular homeostasis and biological functions. After a primary injury of the spinal cord, excessive ROS-induced oxidative damage is closely associated with neuroinflammation, excitotoxicity, and cell death, which represent hallmarks of the secondary damage cascade. Based on these findings, therapeutic strategies targeting oxidative stress and relevant signaling are expected to be effective in mitigating secondary injury, as documented in preclinical experiments. In the last decade, accumulating *in vitro* and *in vivo* studies have indicated that sirtuins are promising therapeutic targets in SCI. However, these therapies have been studied under certain *in vitro* conditions or in animal models of SCI that are not fully representative of the pathophysiology of SCI in humans. In addition, most of the studies did not involve transgenic animal models, which could have provided stronger evidence that antioxidant protection in SCI is dependent on sirtuins. Therefore, to better utilize sirtuins against oxidative stress in the treatment of SCI, more extensive studies in animal models and human clinical trials are needed to validate these therapeutic approaches.

## Figures and Tables

**Table 1:** Basic characteristics of sirtuins and the potential mechanisms in SCI.

Class	Sirtuins	Intracellular distribution	Activity	Function	Potential mechanisms
I	SIRT1	Nucleus, cytoplasm	Deacetylase	Oxidative stress, inflammation, apoptosis, autophagy, metabolism	SIRT1 activated by resveratrol inhibits neuronal apoptosis in SCI rats, reduces tissue damage, and promotes motor function recovery by activating autophagy mediated by the SIRT1/AMPK signaling pathway [131] SIRT1 activated by resveratrol promotes sonic hedgehog signaling to exert antioxidant and anti-inflammatory effects to inhibit fibrous scar formation after SCI [135] MLN4924 significantly attenuates oxidative stress and neuronal cell death by regulating SIRT1 expression during spinal cord ischemia-reperfusion injury [30] *miR-448* inhibits neuronal apoptosis and improves neurological function by upregulating SIRT1, thereby alleviating spinal cord ischemia-reperfusion injury [142] SIRT1 exerts neuroprotective effects by downregulating Wnt/*β*-catenin signaling to inhibit microglia activation, thereby reducing inflammation and cellular stress in the early stages of SCI [144] The SIRT1/Nrf2 pathway in astrocytes can be activated by NFAT5, which exerts antioxidative stress effects against oxygen-glucose-serum deprivation/restoration damage [145].
	SIRT2	Nucleus, cytoplasm	Deacetylase	Cell cycle, oxidative stress, inflammation	SIRT2 promotes the differentiation of ependymal stem cell into neurons after SCI by increasing the deacetylation of stable Ac-*α*-tubulin in microtubules to improve neural recovery [147]
	SIRT3	Mitochondria	Deacetylase	Oxidative stress, apoptosis, autophagy, metabolism	SIRT3 and PGC-1*α* protect rat spinal cord motor neurons from mutant SOD1(G93A)-induced mitochondrial fragmentation and neuronal cell death by maintaining mitochondrial dynamics [148]
II	SIRT4	Mitochondria	Deacetylase, ADP-ribosyl transferase	Inflammation, oxidative stress, metabolism	SIRT4 inhibits the antineuroinflammatory activity of regulatory T cells infiltrating in the traumatically injured spinal cord by suppressing the AMPK signaling pathway [149]
III	SIRT5	Mitochondria	Deacetylase, desuccinylase, demalonylase	Oxidative stress, apoptosis, metabolism	SIRT5 plays a major role in PKC*ε*-mediated neuroprotection against cortical degeneration and neural cell death following cerebral ischemia [150]
IV	SIRT6	Nucleus	Deacetylase, demyristoylase, depalmitoylase, ADP-ribosyl transferase	DNA repair, oxidative stress, apoptosis, autophagy, inflammation, metabolism	SIRT6 could act as a protective factor to attenuate SCI by inhibiting inflammation, oxidative stress, and cell apoptosis [152]
	SIRT7	Nucleolus	Deacetylase	Oxidative stress, apoptosis, rRNA transcription	SIRT7 may protect neurons from oxygen-glucose deprivation and reoxygenation-induced damage by regulating the p53-mediated proapoptotic signaling pathway [154]

Abbreviations: SIRT: sirtuin; SCI: spinal cord injury; AMPK: AMP-activated protein kinase; phosphatase and tensin homolog: PTEN; PKC*ε*: protein kinase C epsilon; PGC-1*α*: peroxisome proliferator-activated receptor-*γ* coactivator-1*α*.

## Abbreviations

SCI: Spinal cord injury
MP: Methylprednisolone
ROS: Reactive oxygen species
NAD: Nicotinamide adenine dinucleotide
SIRT: Sirtuin
SOD: Superoxide dismutase
CAT: Catalase
Sir2: Silent information regulator 2
PGC-1*α*: Peroxisome proliferator-activated receptor-*γ* coactivator-1*α*
FOXO: Forkhead box O
NF-*κ*B: Nuclear factor-kappa B
OA: Osteoarthritis
HIF-1*α*: Hypoxia-inducible factor-1*α*
IDH2: Isocitrate dehydrogenase 2
MnSOD: Manganese superoxide dismutase
Nrf2: Nuclear factor erythroid 2-related factor 2
CNS: Central nervous system
AMPK: AMP-activated protein kinase.
